# Evaluation of the effectiveness of robotic gait training and gait-focused physical therapy programs for children and youth with cerebral palsy: a mixed methods RCT

**DOI:** 10.1186/s12883-016-0582-7

**Published:** 2016-06-02

**Authors:** Lesley Wiart, Rhonda J. Rosychuk, F. Virginia Wright

**Affiliations:** Department of Physical Therapy, Faculty of Rehabilitation Medicine, University of Alberta, 2-60 Corbett Hall, Edmonton, Alberta T5G 2G4 Canada; Glenrose Rehabilitation Hospital, Edmonton, Alberta Canada; Department of Pediatrics, Faculty of Medicine & Dentistry, University of Alberta, Edmonton, Alberta Canada; Edmonton Clinic Health Academy (ECHA), Rm 3-524, 11405 87 Avenue NW, Edmonton, Alberta T6G 1C9 Canada; Bloorview Children’s Hospital Foundation, Bloorview Research Institute, Rm4W-270, 150 Kilgour Rd, Toronto, Ontario M4G 1R8 Canada; Department of Physical Therapy and Rehabilitation Sciences Institute, University of Toronto, Toronto, Canada

**Keywords:** Cerebral palsy, Robot assisted gait training, Physical therapy, Motor skills

## Abstract

**Background:**

Robot assisted gait training (RAGT) is considered to be a promising approach for improving gait-related gross motor function of children and youth with cerebral palsy. However, RAGT has yet to be empirically demonstrated to be effective. This knowledge gap is particularly salient given the strong interest in this intensive therapy, the high cost of the technology, and the requirement for specialized rehabilitation centre resources.

**Methods:**

This is a research protocol describing a prospective, multi-centre, concurrent mixed methods study comprised of a randomized controlled trial (RCT) and an interpretive descriptive qualitative design. It is a mixed methods study designed to determine the relative effectiveness of three physical therapy treatment conditions (i.e., RAGT, a functional physical therapy program conducted over-ground (fPT), and RAGT + fPT) on gait related motor skills of ambulatory children with cerebral palsy. Children with cerebral palsy aged 5–18 years who are ambulatory (Gross Motor Function Classification System Levels II and III) will be randomly allocated to one of four treatment conditions: 1) RAGT, 2) fPT, 3) RAGT and fPT combined, or 4) a maintenance therapy only control group. The qualitative component will explicate child and parent experiences with the interventions, provide insight into the values that underlie their therapy goals, and assist with interpretation of the results of the RCT.

**Discussion:**

n/a.

**Trial Registration:**

NCT02391324 Registered March 12, 2015.

## Background

Cerebral palsy is the most common cause of childhood physical disability, affecting 2.0–2.5 in 1000 children [1]. It represents a group of disorders of movement and posture with impairments (e.g., muscle weakness, decreased selective motor control, alterations in muscle tone, and impaired postural control) that collectively affect functional mobility. Methods of mobility are highly variable in children with cerebral palsy. Approximately 65 % of children with cerebral palsy use minimal or no assistive devices (leg braces, walkers, and/or wheelchairs) to walk (i.e., Gross Motor Function Classification System [GMFCS] Levels I and II) [2] while those in GMFCS Levels III - V require varying degrees of bracing, walkers, or wheelchairs for mobility. Walking abilities can change during the life course; young adults who were ambulatory as children may lose the ability to walk in early adulthood due to joint pain and walking inefficiency [3].

Walking has well-recognized physiological and functional benefits including prevention of muscle contractures [4], maintenance of bone density [5], and enhanced cardiovascular fitness [6]. Effective mobility, which can include ambulation or the use of assistive technology such as powered wheelchairs, confers psychological benefits by fostering children’s abilities to interact with peers and explore their environments [7]. Walking is often emphasized because of the dominant societal beliefs about the symbolic value of walking that is associated with normalcy and reduction of the social stigma of disability [8].

Partial bodyweight support treadmill training (PBWSTT) has recently received attention to improve walking patterns and endurance of children with cerebral palsy [9]. This training facilitates repeated, partially controlled step-taking with a sling giving body weight support to allow greater freedom of movement. Repeated active movement is aligned with motor learning theory currently popular in rehabilitation practice as a means of inducing neuroplastic changes in the brain [10]. Motor learning approaches emphasize movements that involve affected neural networks for motor control through high intensity practice of motor tasks [10], feedback on performance through trial and error, and active engagement of the child/adult in producing and refining movement [11]. There is some evidence that PBWSTT may promote improvements in temporal aspects of gait, walking speed, and gross motor abilities in children in GMFCS Levels II to IV [12]. However, it is labour intensive since therapists need to provide extensive physical support including assistance with the reciprocal leg movements. This limitation has sparked international interest in the potential of robot assisted gait training (RAGT) devices as a better approach to gait training in people with neurologic conditions.

RAGT devices such as the Lokomat® support an adult or child upright on a treadmill while using robotics to move his/her legs to simulate walking. The robotic device facilitates inter-limb co-ordination and gait cycle timing and provides variable degrees of body weight support and guidance, both of which can be decreased as the child progresses. The adjustable weight support allows the child to train at various walking speeds [13]. The biofeedback and virtual reality system (using an avatar that reflects force and movement generated by the child) gives a motivational environment with real-time feedback on force and position. RAGT is also purported to be more cost-effective than PBWSTT as far as personnel and labour [14] due to lower need for manual work by therapists. Early research evaluating the use of RAGT devices (usually the Lokomat®) in adults post stroke or spinal cord injury seemed promising [15], however recent RCTs have not found RAGT to be more effective than regular, gait-focussed physiotherapy [9].

There have only been a few studies evaluating the effectiveness of RAGT with children and youth with cerebral palsy. Initial research was conducted with ambulatory individuals aged 4 to 20 years with cerebral palsy. In these small sample, one-group, pre-post intervention studies [16–20] participants showed improvements in gross motor skills (as measured by the Gross Motor Function Measure [GMFM] [21]), gait velocity and endurance [16, 22], and gains were maintained for 6 months [18]. Participants improved equally on the GMFM Stand and Walk Dimensions (i.e., mean gains about 5 points in each after 12 sessions given over 3 weeks), suggesting an added effect on postural stability for standing skills [20]. Appreciable changes in motor performance were achieved after participation in a short but highly intensive Lokomat® program (i.e., 3 to 4 weeks, total of 12 to 16 sessions) [20, 22]. GMFM Walk Dimension improvement was linked with total distance and time walked on the Lokomat® (*r* = -0.75, *p* < 0.001), [20] suggesting a dose dependency. However the lack of a control group in these studies precludes firm conclusions about the efficacy of RAGT. A recent small RCT with 52 children with cerebral palsy (GMFCS II/III) demonstrated no advantage of RAGT over a physical therapy program for walking speed or range of motion [23], however the authors cautioned against making firm conclusions due to several study limitations. Research in this area is generally comprised of studies that are methodologically weak (i.e., one group pre-post test designs, small sample sizes that limit statistical power and often lack clear descriptions of therapy protocols) [9]. Outcome measurement is often limited to the GMFM, which, provides information on foundational motor skills, but does not measure impacts on functional abilities and participation.

Additional criticisms of RAGT include the inability of current RAGT systems to replicate the real-world demands of overground walking [9]. For example, visual spatial and optical inputs differ and the consistent pace of the device does not offer the opportunity for training temporal aspects of gait (e.g., timing of hip flexion, swing, knee extension). Individuals with cerebral palsy may walk more effectively using deviations from the timing of typical gait patterns. The individual’s reliance on the treadmill and/or robot to create the steps may be too passive thus is not consistent with the ‘real world’ demands of walking. Finally, prolonged focus and extreme efforts towards walking may take away from other important childhood activities and may not be the most efficient way to increase participation [24]. This line of critical thinking requires therapists to be mindful about the role of walking in cerebral palsy rehabilitation, to seek a full understanding of the impact of walking therapies, and to understand the values and perspectives of families in regards to walking especially as new and potentially compelling ‘high tech’ options become more widely available.

### Methods/Design

**Fig. 1:**
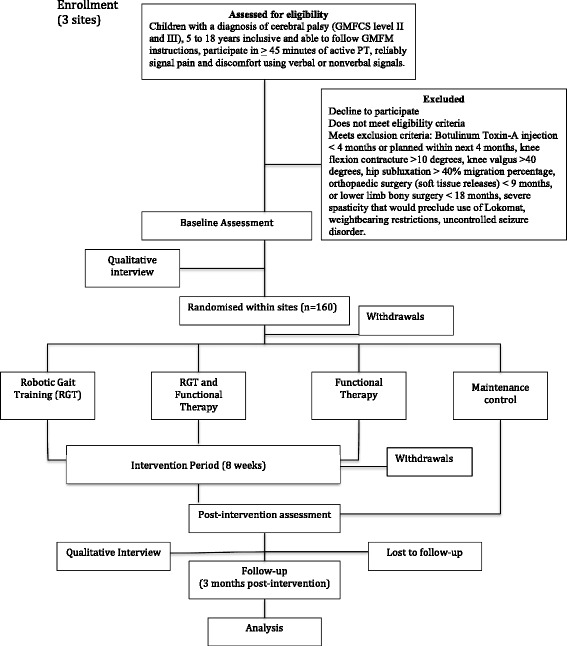
Consort flow chart

This trial is a concurrent, mixed methods study [25]. Specifically, the quantitative arm is a multi-centre RCT with four groups (2^2^ factorial design, i.e., RAGT absent/present, fPT absent/present) with two periods of post-intervention assessments (immediate and 3 months later) (See Fig. 1 for Consort flow diagram). The RCT is linked with an interpretive descriptive [26] qualitative study arm. Methods for the quantitative and qualitative components are described separately.

### Research questions

i) What is the comparative effectiveness of RAGT and a functional therapy program for improving gait-related motor skills of ambulatory children and youth with cerebral palsy?
ii) Does combining RAGT and functional therapy result in greater improvements in gait-related skills of ambulatory children and youth with cerebral palsy than RAGT or functional therapy alone?
iii) What are families’ experiences with the interventions and perceptions of outcomes, and what are the associated implications for interpretation of the RCT results and use of RAGT and functional therapy?

### Randomized controlled trial design

#### Inclusion criteria

1. Children with a diagnosis of cerebral palsy
2. GMFCS level II or III
3. Ages 5 to 18 years
4. Able to follow GMFM testing instructions, and to participate in a minimum of 45 min of active PT
5. Able to reliably signal pain, fear and discomfort using verbal or nonverbal signals

#### Exclusion criteria

1. Botulinum Toxin (Type A) injection < 4 months or planned within next 6 months,
2. Knee flexion contracture > 10°
3. Knee valgus >40°
4. Hip subluxation > 40 % migration percentage
5. Orthopaedic surgery (soft tissue releases) within the last 9 months
6. Lower limb bony surgery < 18 months
7. Severe spasticity that interferes with use of RAGT device
8. Weight bearing restrictions
9. Seizure disorder not fully controlled by medication.

### Sample size

The sample size of 144 children represents 9 children per age group (<13y; 13 to 18y), GMFCS strata, and intervention combination. An additional 16 participants will be recruited to accommodate an estimated 10 % drop out rate (144/.9) for a total of 160. The 10 % drop out rate was based on the current 7 % drop out rate in the feasibility trial conducted at Holland Bloorview. Sample size calculations were based on pre- to post-intervention change on the primary outcome (Gross Motor Function Measure-66) (GMFM-66) [21] and a two-factor factorial design (F tests). For the GMFM-66 change score, assuming a Type I Error of 0.05 and 36 patients per group, the study will have 85 % power to detect an effect size of 0.25 for the RAGT group, 0.25 for the fPT group, and 0.25 for the interaction. Assuming a standard deviation (SD) of subjects of 10, these effect sizes correspond to an actual SD among appropriate means of 2.5. For the GMFM-66, Dimensions D and E, mean change scores of 5.3 (SD = 5.6) and 5.9 (SD = 7.1) have been reported after Lokomat® training [20] and 4.6 (SD = 7.1) for PT [27]. These numbers correspond to effect sizes of 0.9 (GMFM Dimension D), 0.8 (GMFM Dimension E) for the Lokomat®, and 0.6 (GMFM-66 score) for PT. However, a change of 3 points on the GMFM-66 is considered a clinically important difference [28]. Thus we have amplified our power to detect a small effect size (0.25) among groups. PASS [29] was used to calculate sample size. Recruitment will take place at the three sites Holland Bloorview (Toronto, Canada), Glenrose Rehabilitation Hospital (Edmonton, Canada) and the Rehabilitation Institute of Chicago (Chicago, U.S.) over a period of 4 years.

### Randomization

Following the screening assessment, participants will be randomly allocated to one of the four groups using computer-generated random sequence with varied block sizes to prevent randomization pattern prediction by investigators and ensure balanced group sizes. Age (<13 y; 13 to 18 y), GMFCS level (II and III), and site (Holland Bloorview, Glenrose Rehabilitation Hospital, Institute of Chicago) will be used as stratification variables to ensure group balance. The random sequence will be uploaded into the RedCap randomization module [30].

### Blinding

Physical therapist assessors will be blinded to group allocation*.* The three site research assistants will email an independent research assistant (not otherwise connected with the trial) to obtain the group assignment from the randomization module of a RedCap [30] database (as per the randomization schedule) once the child’s baseline assessment has been completed. Group assignment will be conducted several days post initial assessment (parent is informed by phone of the child’s group allocation). The data analyst will be blinded to group allocation. Blinding of child/parent to group is not possible given the nature of the interventions.

### Treatment

There are three intervention groups: 1) RAGT, 2) RAGT + fPT, 3) fPT, and 4) one maintenance therapy control (CONT) arm. All three intervention groups will receive two 50-min sessions per week, separated by 2 or 3 days conducted over 8–10 weeks. This protocol meets the minimum recommended duration of 60 days for intensive interventions (as determined in a meta-analysis of PT treatments in cerebral palsy) [31]. In addition, 3 sessions per week may be very challenging for families and therefore the planned intensity and duration is also based on clinical feasibility.

The LOK and fPT sessions are built on current motor learning theory principles [32] and scoring of extent of their use (treatment fidelity) will be possible via use of the Motor Learning Strategy Rating Scale (MLSRI-20) [33] by an external PT assessor with videos from two sessions per child (4^th^ and 8^th^ sessions) [32]. The prompt scoring of the session video and review by the centre investigator will permit prompt feedback to the treating PT if motor learning strategy use levels do not reach the targeted minimum score of 40 %.

Children in all four groups may continue to participate in ‘maintenance therapy’ (commonly done by children with cerebral palsy between blocks of active therapy) if they are doing so prior to the study. This may include range of motion/stretching and basic isometric strength home program as well as up to 10 min per day of exercise bicycle **or** treadmill **or** general walking practice. Families will be asked to discontinue other active therapy during the trial.

At each site, pediatric physical therapists and physical therapy assistants with expertise working with children with cerebral palsy will be trained to provide RAGT and fPT intervention protocols. Each child will be assigned to a treatment team of two PTs who will share responsibility for the 8 to 10 week intervention phase. The use of a collaborative two-member team is consistent with current models of service delivery in which a physical therapist and physical therapy assistant or second physical therapist share responsibility for a child’s treatment. The team approach also permits maximum scheduling flexibility for the families. Strict guidelines regarding the approaches that may/may not be used have been developed for the fPT and RAGT interventions. Since all three interventions are manualized as well as menu- and goal-based, the consistency of treatment focus/content between PTs is maximized. This is especially important for the fPT intervention due to increased potential for individual physical therapy variation given the wide breadth of treatment options available.

Children in the RAGT and RAGT + fPT groups will also be assigned a physical therapy assistant who will attend each RAGT session to assist with set-up/exit of the child in the Lokomat as well as with integrated use of other equipment, e.g., balls, beanbags. The assistant will not be required during the fPT sessions unless the treating physical therapist determines that their help is required for taller/heavier youth in GMFCS III to ensure therapist and child safety of movement for some or all intervention activities. The extent and duration of physical therapy assistant involvement will be documented in the child’s session log.

### RAGT

Participants will have one fitting visit/acclimatization session before the actual treatment sessions begin. Participants in the RAGT arm will receive two 50-min sessions per week. The study manualized RAGT walking protocol provides methods for progressing/tracking including a 5-min over ground walking session after RAGT to facilitate transfer of motor learning to usual walking devices [9]. The first RAGT walk will be 20 min, increasing, as able, to 45 min plus 5 min of over ground walking at the end [15]. The goal-based RAGT program uses a standardized approach to progressing body weight and guidance support and includes upper body activities while walking to encourage dual tasking and improved posture, and motor imagery practice. All robot settings and activities will be recorded in the session log.

### fPT

Participants will have two 50-min sessions per week. The manualized motor-learning based protocol forms the basis for this intervention. Its focus is on balance (a key issue for children with cerebral palsy that cannot be addressed in the fully supportive RAGT device) and multi-plane gait-based motor skills. Each weekly fPT session will consist of 50 min of active treatment, a ‘dose’ equivalent to time spent in active treatment in the RAGT arm. The treatment program is menu-based. The physical therapist will choose areas that best link with the child’s goals and abilities [34] and document these in the session log. Techniques that focus exclusively on body structure changes will be not be permitted (e.g., inhibitive casting, kinesiotaping, functional electrical stimulation).

### RAGT + fPT group protocol

Participants will alternate between RAGT and fPT sessions for the duration of the 8 to 10 week intervention phase. Sessions will consist of two sessions of RAGT 1 week alternating with two sessions of fPT the following week. RAGT will always commence in week 1. The fPT will build on motor learning principles because the activities will allow the child to practice motor skills in a variety of different activities. The fPT sessions will augment and build on the previous week’s RAGT work, and set the stage for the following week’s RAGT sessions. Techniques focusing on body function/structure changes will be prohibited.

### Monitoring co-interventions

Maintenance therapies such as home stretching and strengthening routines can be continued for all four groups throughout the study because these therapies have questionable efficacy [35–37] and will likely be equally used across all four groups as they are common PT recommendations. Mobility-based active therapy must be discontinued ≥ 2 weeks prior to baseline assessment. Throughout the 8–10 week intervention period and the 3-month follow-up period, parents of children in all four groups will be asked to report about other therapies received or physical activities participated in during the week. Use of other gross motor interventions will also be tracked by the treating physical therapists.

### Outcomes

All study outcomes will be measured pre-/post-intervention (< 10 days pre-intervention and post-completion), and at 3 m follow-up (+/- 10 day window). Trained pediatric physical therapists with pediatric experience will be trained to conduct the assessments. Assessors will be assigned to a child. While inter-rater reliability of all of the selected measures is good to excellent, use of the same assessor will support a smaller minimum detectable change. This sensitivity is particularly important since the sample size was based upon a small (but clinically important) effect size. Prior assessment data will not be available to the assessor at the follow-up assessments. Assessments will be video-recorded and a random sample of 20 % of the assessment’s video-recordings will be scored by an independent assessor who will not be aware of assessment sequence order. This double scoring will be done through the study to flag any scoring issues and allow remedial action.

#### Primary outcome

The primary outcome measure is the GMFM-66 [21]. It has strong validity and responsiveness with children with cerebral palsy and has been used in prior RAGT studies. The GMFM-66 evaluation will be limited to Dimensions D (Stand) and E (Walk/Run/Jump).

#### Secondary outcomes

Secondary outcomes include higher level gross motor functioning (the Challenge Module) [38] for children in GMFCS level II, walking capacity [39, 40], gait quality [41] individualized goal attainment scores [42, 43], balance [44, 45], quality of movement [46], functional abilities [47], physical activity levels, self-efficacy for physical activity [48], participation (PEM-CY) [49], and quality of life [50, 51]. A list of included outcomes and associated measures are included in Table 1.

**Table 1:** Outcome measures

Outcome	Outcome measures
Gross motor abilities	GMFM-66 (Dimensions D- Stand & E – Walk/Run/Jump) [21] Challenge Measure (GMFCS level II) [38] and GMFM Dimensions D and E with aids and orthoses (GMFCS III) [21]
Walking capacity/gait	1- [39] and 6-Minute Walk Test [40], Bloorview Barefoot Gait Assessment (scored from video) [41]
Individualized goal attainment	Canadian Occupational Performance Measure (COPM) [42] and Goal Attainment Scale (GAS) [43]
Standing and Walking Balance	Pediatric Balance Scale [44], Quality FM (Stability from GMFM-66 video) [46], Activities Balance Confidence Scale [45]
Functional abilities	PEDI-CAT [47]
Physical Activity levels	Accelerometry (5 days)
Physical Activity Self-efficacy	Self-Efficacy for Physical Activity [48]
Participation	Participation and Environment Measure for Children and Youth (PEM‐CY) [49]
Quality of life	KidScreen [50] and Students’ Life Satisfaction Scale (SLSS) [51]

### Statistical analysis

Data will be described (e.g., means, standard deviations, frequencies) for each intervention group and each stratification variable. Graphical summaries will include mean plots and boxplots. Change scores (post minus pre, follow-up minus post) will be summarized for each outcome. For each change score and outcome, an ANOVA for the two-factor factorial design will test the effect of each factor (RAGT, fPT) and their interaction (RAGT*fPT) on mean change score. Confidence intervals (95 % CIs) will be reported for the mean of each intervention group. Further, mixed-effects multiple linear regression models will be developed for each outcome with centre as a random effect, centre by intervention as an interaction (to assess centre effect), and other important variables (e.g., age and GMFCS level) as covariates. Variables will be dropped from the model one at a time if *p* > 0.05, and residual diagnostics will assess model fit. This modeling will allow us to assess the effect of the interventions in the presence of important variables that were not balanced across intervention groups by randomization and also can easily deal with incomplete observation times. All main analyses will be based on intent-to-treat with secondary analyses of those with >80 % adherence to their intervention. R [52] will be used for statistical analysis by a data analyst blinded to intervention group.

### Data and Safety Monitoring (DSMB)

An independent DSMB will assess any reports of adverse events and will recommend to the researchers if the trial should continue, be modified or stopped. The DSMB will consist of three representatives from Toronto, Edmonton and Chicago. Teleconferences will be scheduled annually.

### Interpretive description (Qualitative Component)

While RCTs are the gold standard for evaluating cause and effect relationships between interventions and patient outcomes [53] there is growing recognition that a broader paradigmatic view of research methodologies is necessary since RCTs do not serve well in the analysis of the complex descriptions of human perspectives and experiences [54–57]. Knowing *why* interventions do or do not work is as important as knowledge of effectiveness if interventions are to be successfully transferred into ‘real world’ clinical settings [53, 58]. Rich contextual information from qualitative research can provide insight into how patient values and previous occurrences affect their experience with the interventions, their adherence to the study protocols, their impressions about the importance of the outcomes achieved, and the reasons why they choose to participate or not in clinical trials [54–57].

The three **objectives** of the concurrent qualitative component are to explicate:

1. Child and parent experiences with the trial interventions and the values and previous experiences that shape their perceptions.
2. The mobility related outcomes that are important to families and factors that influence these views.
3. Child and family values, experiences and contextual factors that influenced participation in the trial, including the follow-up period.

### Design

Interpretive description [59], a methodology designed for conducting rigorous qualitative research within the health professions, will be the framework for the qualitative component of this study. Interpretive description is focused on “generating new knowledge pertaining to the subjective, experiential, tacit and patterned aspects of human health experience… so that we have sufficient contextual understanding to guide future decisions that will apply evidence to the lives of real people” [26]. It provides a ‘design logic model’ for qualitative studies so that the results are meaningful and applicable to clinical practice.

### Sample selection

Since the goals of parents and children may differ [60] and be informed by different values regarding the importance of walking [61], both will participate. The inclusion of parents and children will allow us to gain a greater understanding of family dynamics and shared understandings [62] that affect their experiences in the trial. We will invite a subset of child-parent dyads from each of the active interventions in the RCT and seek maximum variation in this purposive sample by ensuring an equal number of children in the two age groups (i.e., under and over 13) and both GMFCS levels, as well as a diversity of cultural and socioeconomic status (critical for objective #2). We will recruit families from all three sites since factors that affect trial participation may vary between provinces and centres based in Canada and the United States. In addition, parents of children who were eligible but declined to participate in the RCT will be invited to participate in the qualitative component to address objective #3. The estimated sample size is based on theoretical understanding of the complexity and variability of the data. We anticipate that a sample of 18 RCT participant child-parent dyads (6 dyads from each site) and 3 parents from each site who declined participation in the RCT will be adequate to address the three objectives of the qualitative component. Our estimate is based on similar qualitative research with families with children with cerebral palsy [63–65] and is considered to be a relatively large sample for this type of research [26].

### Data collection

Individual interviews with parents (and their children for those in the RCT) will be conducted. Parents will participate in 45–60 min semi-structured, individual interviews conducted by one member of the research team. Participating parents of children in the RCT will be interviewed at 2 points within the trial (Fig. 1): i) after identifying their individualized goals, prior to receiving the intervention, and ii) within 1 month of intervention completion.

Children from the RCT will participate in individual interviews at the end of their intervention. While interviewing children can pose some logistical challenges, if adapted techniques are employed, children have the potential to share rich narratives [62, 66]. A customizable “tool box” of age-appropriate child-friendly techniques [61] including photographs and comic captioning, vignettes, and sentence starters will be used in a 30–45 min semi-structured interview with the child without the parent present. The use of “concrete materials” in interviews with children has been found to improve the quality and depth of the interview exchange [67].

### Data management and analysis

Interviews will be digitally audio-recorded, transcribed verbatim by a professional transcriptionist, de-identified and imported into NVivo for data management. Data sources will be digital recordings, transcribed text of all interviews, and field notes created by the interviewers. The data management strategy described by Knafl [68] will be used to analyze the data. Two researchers will collaboratively identify general coding categories. Transcripts will be analyzed as they are transcribed to ensure that the emerging results inform the concurrent theoretical sampling and data collection process [69]. The researchers will meet to establish consensus on the coding. Data will be transferred to index cards and organized by general codes. The two researchers will identify subthemes and the RA will conduct the remaining coding of excerpts into subthemes in NVivo [70]. This process is recommended for interpretive descriptive studies [59] as it involves immersion in the data prior to any specific coding and emphasizes theorizing, synthesizing and re-contextualizing [26]. Field notes will not be coded but will aid data interpretation, as they will contain interviewer impressions and observations during the interviews.

### Enhancing credibility

i) **Methodological triangulation **- The use of multiple methodologies or data sources will add rigor, depth, complexity and richness to any research study [71]. The results of this concurrent qualitative component will provide essential context and meaning to the interpretation of the change scores from the RCT.
ii) **Maximal variation in sampling **- Ensuring variability on the factors that likely influence family perspective such as child age, GMFCS level, treatment condition and site will enhance credibility of the data. Lack of attention to this variation may result in inaccurate claims about groups that were not included in the sample [72].
iii) **Audit trail **– Recording of methodological decisions and their rationale as made throughout the qualitative study will let us include reasoning in the final report so that the consumers can judge adequacy of decision-making [26].
iv) **Peer debriefing **– A summary of the qualitative data analysis process will be reviewed and discussed by all members of the research team mid-analysis stage (i.e., once general themes are identified) and after identification of sub-themes. Team members not involved directly in the analysis will be encouraged to ask critical questions about methods, decisions and interpretation to facilitate reflection among the team members conducting the analysis. Peer debriefing sessions will be documented [53].

## Discussion

It is critical to know if children with cerebral palsy benefit from RAGT more than over-ground walking training programs and maintenance therapy alone. The results of this trial will provide important insight into the relative effectiveness of RAGT and functional physical therapy. We will measure a broad range of outcomes that could potentially be affected by RAGT, and we anticipate that the trial will provide information needed to guide clinical practice related to RAGT for children with cerebral palsy.

RAGT is currently used clinically at the Edmonton and Chicago sites and is used only for research in Toronto. We anticipate that we may encounter some challenges at recruitment at the two sites where RAGT is used clinically. Our primary concern is related to possibility of parents’ preference for clinical use of RAGT over the possibility of being randomly allocated to a control group. To mitigate this risk, we may inform parents of group allocation following the screening assessment and then will allow some flexibility in the timing of the baseline assessment. This flexibility will allow families to schedule according to group allocation. They may decide to access clinical RAGT after the control period and during the summer months, when many families request therapy. In addition, children in the control group will be able to access their choice of therapy upon completion of the study to mitigate the risk of drop outs due to allocation to the control group.

The qualitative component will enhance the interpretability of the quantitative data through data triangulation [73]. In addition, the inclusion of a qualitative component in this RCT will serve three key purposes. Firstly, it will provide insight into the subjective experience of children and their parents with the trial interventions and how their values regarding quality of gait and previous therapy may have shaped those experiences. Understanding these experiences is highly relevant to the implementation of gait-related interventions in clinical practice to ensure that children and parents are engaged, able and motivated to participate in therapy. Research in this area is lacking.

Secondly, the qualitative findings will provide valuable information about the outcomes of mobility interventions that are important to families. While RAGT replicates a ‘typical’ gait pattern with the hope that this will transfer into overground walking, some research suggests that compensatory movement patterns may be more efficient for individuals with cerebral palsy [74]. Indeed, there is a lively philosophical debate regarding the assumption that individuals with disabilities always strive to appear or feel more ‘normal’ [75]. While dominant cultural views and traditional approaches in pediatric rehabilitation have focused on quality of gait, many individuals with disabilities, therapists and researchers insist that improved functional abilities and participation in meaningful activities and social roles are more important outcomes. These may be achieved using alternate methods of mobility, atypical gait patterns, or compensatory approach to rehabilitation. Thus, in addition to understanding the impact of RAGT impact on gait outcomes, functional mobility and participation in meaningful activities, it is important to elucidate the factors that influence child and parent goals related to mobility.

Finally, the qualitative component will allow us to examine reasons families chose to participate or not in the trial. For example, previous research suggests that parents may want to pursue sophisticated interventions for their children because they align with their conceptualization of good parenting and the importance of ‘doing something [61]. It is also possible that contextual factors such as challenges with travel, time commitment, and managing family life or conflict with values around walking may preclude some families’ participation. This information is useful regarding the feasibility of implementing the interventions in clinical practice and for interpreting trial outcomes.

## Abbreviations

CONT: control group
fPT: functional physical therapy
GMFCS: gross motor function classification system
GMFM: gross motor function measure
MLSRI: motor learning strategy rating instrument
PBWSTT: partial body weight supported treadmill training
RAGT: robot assisted gait training
RCT: randomized, controlled trial
SD: standard deviation
